# Improved quantification of photoacoustic biomarkers through residual-based noise masking^☆^

**DOI:** 10.1016/j.pacs.2026.100880

**Published:** 2026-09-10

**Authors:** David Qin, Xinyue Huang, Scott J. Schoen, Stanislav Emelianov, Richard R. Bouchard

**Affiliations:** a Wallace H. Coulter Department of Biomedical Engineering, Georgia Institute of Technology and Emory University School of Medicine, Atlanta, GA, 30332, USA; b School of Electrical and Computer Engineering, Georgia Institute of Technology, Atlanta, GA, 30332, USA; c Department of Imaging Physics, The University of Texas MD Anderson Cancer Center, Houston, TX, 77030, USA; d The University of Texas MD Anderson Cancer Center UTHealth Graduate School of Biomedical Sciences, Houston, TX, 77030, USA

**Keywords:** Noise, Photoacoustic imaging, Quantitative, Masking, Biomarker, Sensitivity

## Abstract

Spectroscopic photoacoustic (PA) imaging enables non-invasive, label-free estimation of *in vivo* biomarkers such as tissue oxygen saturation (SO_2_). Quantitative accuracy is limited by low signal-to-noise ratio (SNR) in deeper regions from fluence attenuation and heterogeneous absorber concentration. In these regions, electronic noise can dominate PA spectra, yielding physiologically-plausible but spurious SO_2_ estimates. Since biomarkers are reported as values averaged over a region-of-interest (ROI), including noise-dominated pixels introduces systematic bias and reduces sensitivity to physiological changes. We present a pixel-wise noise masking framework: at each pixel, a linear least-squares residual compares the measured PA spectrum and a reference noise spectrum, and residual statistics within a signal-free ROI define a masking threshold. Unlike denoising, this approach excludes unreliable pixels prior to ROI-based estimation without modifying measured signals. Phantom and *in vivo* experiments demonstrate improved sensitivity to SO_2_ dynamics following noise masking, highlighting the need for robust noise exclusion in quantitative PA imaging.

## Highlights

• Electronic noise can dominate deep PA spectra, biasing ROI-averaged biomarkers.
• A normalized spectral residual measures each pixel’s similarity to a noise reference.
• A statistical threshold masks noise pixels prior to ROI biomarker estimation.
• Masking restores sensitivity to biomarker changes in phantom and *in vivo* data.
• The approach may generalize across spectroscopic PA imaging for many biomarkers.

## 1 Introduction

Photoacoustic imaging has seen increasing promise for biomedical applications due to its combination of optical molecular contrast, sub-millimeter resolution, and enhanced imaging depth from acoustic detection. Photoacoustic imaging (PA) relies on pulsed laser excitation of tissue chromophores, causing transient thermoelastic expansion that generates broadband acoustic waves; these propagate to the tissue surface and are detected and beamformed using conventional ultrasound hardware [1]. PA imaging is commonly combined with simultaneous B-mode ultrasound imaging (US), providing a spatially co-registered anatomical image.

When used with multiple laser wavelengths, PA imaging extends to spectroscopic PA imaging (sPA), enabling quantification of various chromophores such as endogenous absorbers (e.g. hemoglobin, melanin, collagen), exogenous agents (e.g. dyes or nanoparticle contrast agents), and even cells labeled with contrast agents *in vivo* [2], [3], [4], [5], [6], [7]. sPA imaging has been extensively studied for non-invasive and label-free estimation of clinically relevant biomarkers such as lipids, collagen, and hemoglobin [8], [9].

While noise suppression has been widely studied [10], [11], [12], [13], the consequences of including noise-dominated pixels in ROI-based biomarker estimation have received comparatively little attention in the PA literature. PA imaging is inherently signal-limited, particularly at depth, due to exponential decay of optical fluence and, to a lesser extent, acoustic attenuation [14], [15], [16], [17]. Together with heterogeneity in the concentration of optical absorbers, regions of the image may have low signal-to-noise ratio (SNR), wherein electronic noise dominates the measured spectrum. After spectral unmixing, such noise-dominated pixels may yield physiologically plausible but erroneous biomarker estimates. Because clinical imaging workflows commonly report biomarkers as mean values within user-defined ROIs [18], [19], [20], inclusion of low-SNR pixels can introduce systematic bias and reduce sensitivity to physiological changes.

**Fig. 1:**
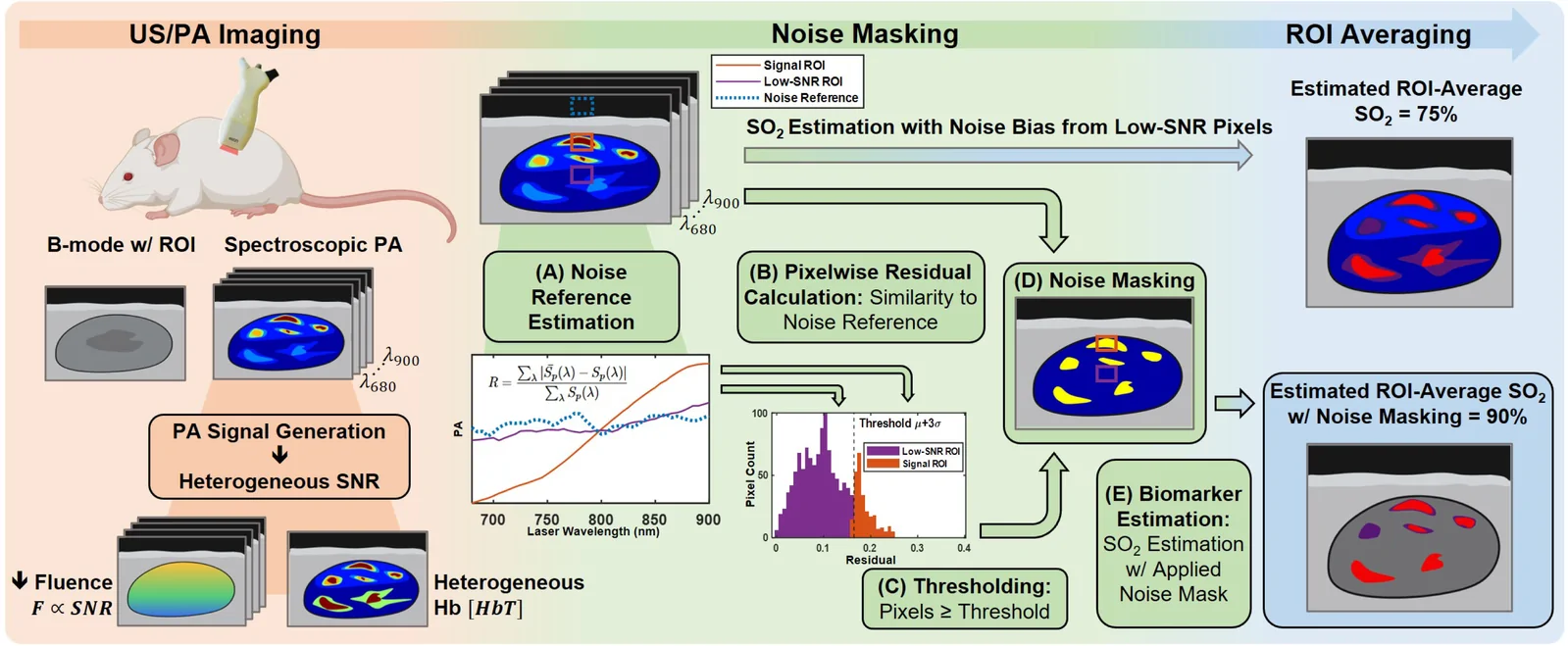
Overview of the residual-based noise masking framework. Spectroscopic photoacoustic imaging inherently has heterogeneous SNR, arising from exponential fluence decay with depth combined with heterogeneous hemoglobin concentration. This produces low-SNR pixels whose PA spectra are dominated by electronic noise, introducing a substantial noise bias if included in ROI-based averages of PA-derived biomarkers. (A) The reference noise spectrum is measured from a signal-free region, such as the near-field standoff. (B) At every pixel, a normalized residual measures the dissimilarity of each pixel’s PA spectrum from the scaled reference noise spectrum. (C) The statistical distribution of residual values in the signal-free region defines a threshold (here μ+3σ). (D) The threshold generates a binary mask that rejects pixels spectrally similar to noise and retains pixels dissimilar to noise (i.e., signal). (E) Applying this mask before unmixing reduces noise-related bias in the ROI-averaged SO_2_ estimate, whereas processing the data without masking incurs a substantial noise bias and sensitivity penalty.

This type of noise bias affects any sPA-derived biomarker, but is particularly consequential for tissue oxygen saturation (SO_2_), which we use here as a representative example. SO_2_ is estimated from the relative concentrations of oxyhemoglobin and deoxyhemoglobin, and serves as a proxy for vascular blood oxygen saturation. Low SO_2_ within tumors — tumor hypoxia — is strongly associated with poor prognosis across a wide range of cancers [21], [22], and a hypoxic tumor microenvironment promotes growth, resistance to the anti-tumor immune response, and tumor angiogenesis [23], [24]. Detecting hypoxia has therefore motivated sustained interest in accurate, non-invasive estimation of SO_2_ in tumors and other pathologies [19], [25].

The approach presented here is conceptually distinct from denoising. Rather than modifying measured signals to suppress unwanted components (e.g., electronic noise), our approach masks noise-dominated pixels to modify the ROI-based estimator of biomarkers. Each pixel’s measured spectrum is compared to a reference noise spectrum obtained from an ROI assumed to be free of signal (e.g., within the near-field standoff). A linear least-squares residual quantifies spectral similarity to noise, and residual values within the noise ROI statistically define the threshold for the noise mask, which is applied to the entire image. Pixels classified as noise-dominated are excluded from subsequent ROI-based biomarker estimation. Our work is distinct from, and complementary with, other efforts that correct for spectral coloring, enhance unmixing, and denoise images [26], [27], [28]. The key innovations of this work are the recognition that noise-dominated pixels introduce systematic bias into ROI-based sPA biomarker estimation, and the development of a residual-based noise masking framework to mitigate this bias without altering the measured sPA signals.

We demonstrate the noise masking approach using the change in tissue oxygen saturation (ΔSO_2_) as a representative example of a clinically relevant biomarker that suffers from noise bias resolvable with our approach. We use ΔSO_2_ specifically because, as a relative estimate, it is less susceptible to spectral coloring than absolute SO_2_ estimates [29], [30]: this allows noise bias to be the focus of our study. We show, in blood-containing phantoms, the impact of a noise bias in ROI-based ΔSO_2_ estimation and demonstrate the improvement in sensitivity provided by noise masking. Finally, we perform an *in vivo* demonstration of noise masking on sPA imaging of an anesthetized rat during an oxygen challenge. These findings highlight pixel-wise noise masking as a complementary step in quantitative photoacoustic imaging and underscore its importance for reliable clinical translation of ROI-based biomarker assessment.

## 2 Methods

This section describes the theoretical basis for the residual-based noise masking framework, the imaging system and data acquisition procedures, the controlled *in vitro* phantom experiments used to characterize and demonstrate the method, and the *in vivo* oxygen challenge experiments used to demonstrate the method under a controlled physiological perturbation.

### 2.1 Calculation of the residual

Noise masking is based on the observation that electronic noise in a PA acquisition arises from the acquisition hardware rather than from laser-tissue interaction, and therefore does not vary with illumination conditions or absorber distribution. As a result, noise exhibits a consistent and characteristic spectral signature throughout the image: spectrally flat when no energy correction is applied, or scaled by the wavelength-dependent energy correction factor when it is used. In either case, this signature is approximately spatially uniform and stable across acquisitions, making it estimable from a signal-free region such as the near-field standoff. This property enables a pixel-wise residual quantifying how closely each pixel’s measured spectrum resembles the noise spectrum, providing a statistical basis for distinguishing noise-dominated pixels from signal.

To estimate the noise spectrum, the mean PA amplitude is measured across a region of interest (ROI) expected to be free of PA signal (e.g., within the near-field standoff region). This yields a reference noise spectrum $S_{n}\left(\lambda \right)$ accurate to the imaging session regardless of whether energy correction has been applied (Fig. 1, A). For each pixel with observed spectrum $S_{p}\left(\lambda \right)$, the noise spectrum is scaled to best match the observed spectrum via least-squares minimization, yielding a scalar coefficient $\overset{ˆ}{c}$ and a scaled noise estimate:

(1) $${\overset{¯}{S}}_{p}\left(\lambda \right)\;=\overset{ˆ}{c}S_{n}\left(\lambda \right)\;,$$

where $\overset{ˆ}{c}$ is obtained by minimizing the sum of squared differences between the scaled noise spectrum and the observed spectrum:

(2) $$\overset{ˆ}{c}=\underset{c}{\mathrm{argmin}}\underset{\lambda }{\sum }{\left[cS_{n}\left(\lambda \right)-S_{p}\left(\lambda \right)\right]}^{2}\;,$$

which has the closed-form solution:

(3) $$\overset{ˆ}{c}=\frac{\underset{\lambda }{\sum }S_{n}\left(\lambda \right)S_{p}\left(\lambda \right)}{\underset{\lambda }{\sum }S_{n}{\left(\lambda \right)}^{2}}\;.$$

The residual R is then defined as the normalized absolute difference between the scaled noise estimate and the observed spectrum:

(4) $$R=\frac{\underset{\lambda }{\sum }\left|\overset{ˆ}{c}S_{n}\left(\lambda \right)-S_{p}\left(\lambda \right)\right|}{\underset{\lambda }{\sum }S_{p}\left(\lambda \right)}\;.$$

Normalizing by $\sum_{\lambda }S_{p}\left(\lambda \right)$ causes R to be dimensionless and reduces its sensitivity to pixel-wise amplitude variations arising from system parameters such as time-gain compensation and transducer element directivity. A pixel whose spectrum closely matches the noise spectrum will produce a residual near zero, while a pixel containing true PA signal will have a spectrum that deviates from the noise reference, yielding a larger residual (Fig. 1, A/B).

To establish a statistically based masking threshold, residual values are computed across an ROI assumed to be void of PA signal, such as the proximal region of the gel standoff. The resulting distribution of residuals within this “noise-only” region is characterized by its mean μ and standard deviation σ, permitting reliable statistical characterization from either a fixed pixel across a sufficient number of temporal frames (≥100 frames; see Section 3) or a sufficient number of pixels within an ROI of a fixed frame (≥500 pixels). For this work, the threshold for the noise mask was set at:

(5) $$R_{T}=\mu +3\sigma \;.$$

We used μ+3σ as our threshold because this corresponds to a theoretical upper-tail probability of approximately 0.14% to retain noise; the less stringent μ+σ and μ+2σ thresholds correspond to approximately 15.9% and 2.3%, respectively. Pixels with residual values below $R_{T}$ are classified as noise-dominated and excluded from subsequent ROI-based biomarker estimation, while pixels at or above the threshold are retained as signal-bearing (Fig. 1, C/D). This framework does not modify the measured PA signals in any way; it operates solely as a binary inclusion criterion applied prior to ROI averaging and is therefore conceptually distinct from denoising approaches.

### 2.2 Imaging system

All experiments in this study utilized a Vevo 2100/LAZR combined US/PA system (FUJIFILM VisualSonics, Toronto, ON, Canada) with an LZ-250 linear-array transducer (256 elements, 23 MHz center frequency). Laser excitation (680 to 970 nm, 45 mJ/pulse, 4-6 ns pulse width, 20 Hz PRF) was provided by an Nd:YAG laser with optical parametric oscillator (Vevo LAZR). Light was coupled to the imaging plane via fiber optic bundles flanking the transducer in the elevational direction. PA imaging was always performed in conjunction with B-mode US imaging, providing spatially co-registered anatomical context.

### 2.3 Noise characterization

To characterize the noise spectrum and estimate the statistical distribution of the residual (Fig. 1, A-B), noise-only data were obtained in a tank of degassed water with laser output blocked (i.e., no photoacoustic excitation) without energy correction or frame averaging, using laser wavelengths from 680–900 nm in 10 nm steps. Acquisition parameters (field-of-view, time-gain compensation (TGC), overall system gain) were set to match those used in phantom and *in vivo* imaging studies, ensuring that the noise characterization reflected the same system state. To assess the temporal and spatial stability of the residual, nine hundred US/PA frames were acquired to provide a reference estimate of the residual statistics in a noise-only acquisition. Against this reference, we determined the minimum number of temporal frames and the smallest spatial ROI required for stable, reproducible residual statistics. Pixel-wise residual means and standard deviations were computed both temporally (across all 900 frames at a fixed spatial location) and spatially (across varying numbers of pixels within a single frame), as described in the Results. Stability was defined as convergence of the normalized residual standard deviation to within 3% of the corresponding reference estimate obtained from the 900-frame acquisition. Beamformed PA amplitude data were used throughout, rather than single-channel RF or IQ data, to maintain relevance with the majority of pre-clinical and clinical US/PA systems.

### 2.4 In vitro phantom experiments

Controlled *in vitro* experiments were performed to demonstrate noise bias in ROI-based SO_2_ estimation and to quantify the improvement from noise masking. Polyethylene (PE) tubes (1.1 mm inner diameter, 1.6 mm outer diameter) were filled with heparinized bovine blood at five target SO_2_ levels: 65%, 72%, 75%, 77%, and 91%. Blood samples at full oxygenation and full deoxygenation were produced chemically by addition of hydrogen peroxide or sodium hydrosulfite, respectively, and then combined in the appropriate ratios to achieve each target SO_2_ level [31], [32]. Tubes were imaged in cross-section in one of two conditions: suspended in degassed water (baseline) or within a tissue-mimicking liquid phantom of degassed water and 8% v/v milk [33]. Imaging was performed with 10-frame averaging with energy correction at wavelengths 734, 760, 800, and 850 nm, which were selected for their spectral sensitivity to oxyhemoglobin (HbO_2_) and deoxyhemoglobin (HHb). Images were acquired at seven different elevational slices separated by 1 mm along the tube axis; data from all seven slices were pooled for statistical analysis as technical replicates.

Data were processed in MATLAB R2024a (MathWorks, Natick, MA, USA). Relative concentrations of HbO_2_ ($c^{{\mathrm{HbO}}_{2}}$) and HHb ($c^{\mathrm{HHb}}$) were estimated using linear least-squares spectral unmixing with reference spectra [34], then SO_2_ was determined as:

(6) $${\mathrm{SO}}_{2}=\frac{c^{{\mathrm{HbO}}_{2}}}{c^{{\mathrm{HbO}}_{2}}+c^{\mathrm{HHb}}}.$$

Residuals were computed using the same four wavelengths. A signal-free region of each multi-wavelength image stack was selected as the noise ROI to determine residual statistics (μ,σ), and the threshold $R_{T}=\mu +3\sigma$ was applied to generate a binary noise mask (Fig. 1, A–D). For estimation of ΔSO_2_ between two imaging conditions, the respective noise mask for each condition was used (Fig. 1, E).

Tube cross-sections were segmented manually from B-mode images, and the resulting contours were applied to co-registered PA-derived SO_2_ images. To characterize how noise bias scales with ROI size, the segmented tube ROI (1.6 mm diameter, matched to the tube outer diameter) was progressively dilated in 0.4 mm increments up to 4.8 mm diameter, extending the ROI into the surrounding signal-free water. Mean and pairwise differences in SO_2_ were computed within each dilated ROI, with and without the noise mask applied.

A one-way ANOVA followed by Bonferroni-adjusted pairwise comparisons was used to identify which pairs of SO_2_ levels were statistically distinguishable ($p_{adj}<0.05$). The number of significantly distinguishable pairs, normalized to the count at baseline ROI size, was used as a measure of sensitivity to SO_2_ differences as a function of ROI size. Reference SO_2_ values were established from unmixed data within tube-only (water bath) images, where spectral coloring is minimal. When necessary, SO_2_ estimates from the tissue-mimicking phantom were reported as apparent values, as spectral coloring precludes accurate absolute SO_2_ measurement at depth [29].

### 2.5 In vivo oxygen challenge experiments

*In vivo* experiments were performed to demonstrate noise masking under a controlled physiological perturbation. One female Sprague-Dawley rat was used; all procedures were approved by the Institutional Animal Care and Use Committee at the Georgia Institute of Technology. The animal was anesthetized with 5% isoflurane delivered via 21% O_2_ gas (balance N_2_) then maintained at 3% isoflurane. Fur was removed from the bilateral thigh regions by shaving, and the animal was allowed to equilibrate at normoxia for five minutes prior to imaging.

US/PA imaging was performed continuously on the skeletal muscle of the thigh with 10-frame averaging at the same four wavelengths as the phantom experiments (734, 760, 800, and 850 nm). To perturb tissue SO_2_ in a known and reproducible manner, a normoxic-to-hypoxic oxygen challenge was performed [35]. Briefly, the carrier gas was rapidly switched from 21% to 10% O_2_ (balance N_2_), maintained for 60 s, then returned to 21% O_2_ for four minutes. This normoxic-to-hypoxic challenge was performed three times, eliciting a measurable and reproducible response. We did this in place of a hyperoxic-normoxic challenge (100% to 21% O_2_) as it does not produce a detectable change in resting skeletal muscle SO_2_ [36], a finding consistent with our own preliminary experiments.

**Fig. 2:**
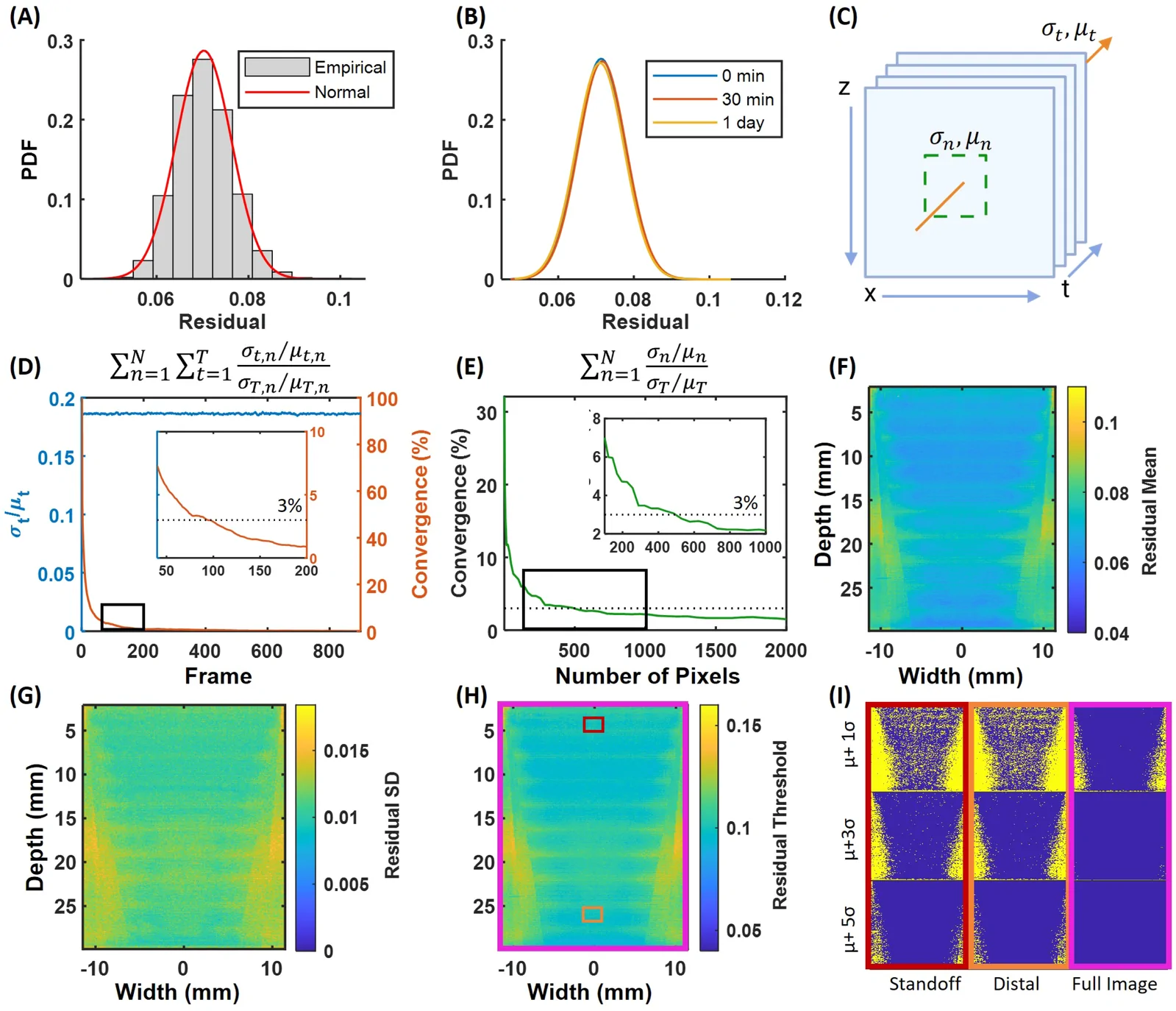
(A) Residual values from a representative ROI were well approximated by a Gaussian distribution, as assessed by comparison of the empirical PDF with a fitted normal distribution. (B) The residual distributions remained similar across imaging sessions at 0, 30 min, and 1 day. (C) Residual convergence was assessed for a 3D (x,z,t) array, with mean μ and standard deviation σ computed temporally for a single pixel (orange) and spatially at a single time point (green). (D) The normalized temporal residual standard deviation converges to within 3% of the 900-frame reference value by approximately frame 100 (orange). (E) Comparable spatial stability is achieved using an ROI of approximately 500 pixels from a single frame (green), corresponding to the minimum ROI size required to achieve the predefined 3% convergence criterion. (F–G) Noise-only residual image showing characteristic IQ-remodulation banding, and its corresponding standard deviation map. (H) Residual threshold at μ+3σ, with proximal, distal, and full-image ROIs indicated. (I) Using each of the three ROIs to estimate residual statistics for the threshold, masks were applied at 1, 3, and 5 standard deviation thresholds, demonstrating progressive noise rejection with increasing threshold.

As before, a signal-free region in the standoff was used to estimate the reference noise spectrum (non-flat due to energy correction) for calculation of the residual and determination of the threshold (Fig. 1, A-C). The noise mask was then applied and PA data were unmixed for HbO_2_ and HHb at every time point to calculate SO_2_ (Fig. 1, D-E). Because spectral coloring confounds absolute SO_2_ measurements in tissue at depth [6], [14], all analyses used ΔSO_2_ (i.e., the change in SO_2_ relative to the pre-challenge baseline). Data from the three oxygen challenge cycles were temporally aligned to the onset of the challenge and pooled for statistical analysis. A one-way repeated measures ANOVA followed by Bonferroni-adjusted pairwise comparisons (α=0.05) was used to determine whether mean ΔSO_2_ differed significantly between the unmasked, masked, and inverse masked (i.e., noise-dominated) pixel subsets.

We then compared the coverage of the mask against a map of pixels whose SO_2_ had undergone statistically significant change over the course of the oxygen challenge. Only pixels containing blood signal are expected to exhibit a statistically significant SO_2_ change, while noise-dominated pixels should not (since noise bias is expected to remain constant before and during the oxygen challenge). For the negative phase of the oxygen challenge (i.e., 21% O_2_ to 10% O_2_), pixel-wise t-tests were performed comparing SO_2_ values from three frames immediately preceding the oxygen challenge against three frames at the end of the negative phase (i.e., the time of lowest oxygen saturation), while for the positive phase (i.e., 10% O_2_ to 21% O_2_), three frames at the lowest oxygen saturation were compared to three frames one minute following that, when SO_2_ had recovered. A one-tailed t-test (α=0.05) was applied at every pixel, with the direction of the test set to detect a decrease in SO_2_ (normoxia to hypoxia) or an increase in SO_2_ (hypoxia to normoxia), consistent with the known direction of the physiological perturbation. The map of the pixels passing this significance criterion was then compared against the noise mask, and spatial colocalization was quantified using the Dice-Sørensen coefficient.

**Fig. 3:**
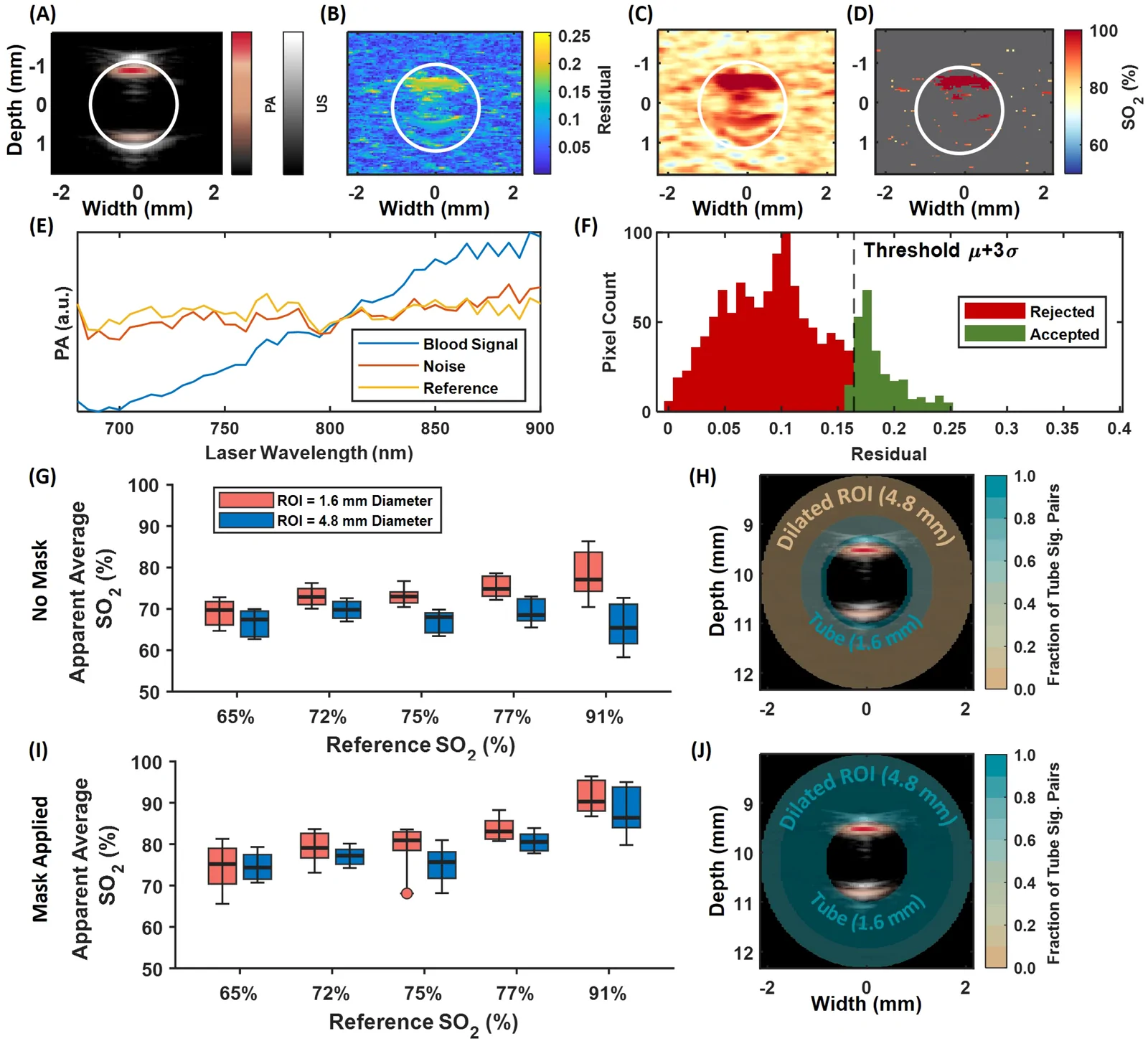
(A) US and PA (800 nm) image of blood in tube phantom, with 1.6 mm tube outer diameter shown as white circle. (B) Residual image showing the region of blood signal with higher values than the background, providing a basis for separation from noise. (C) Unmixed SO_2_ image without masking, showing highly-oxygenated blood (80%–100% SO_2_) surrounded by signal-free regions of the image that unmix to 60%–70%. (D) Application of the residual mask to the SO_2_ image demonstrates effective masking of noise pixels while preserving signal-bearing regions, here showing high SO_2_. (E) The PA spectrum of blood differs substantially from the reference noise spectrum, whereas a noise region adjacent to the tube phantom shows minimal difference. (F) Histogram of residual distributions at the tube, showing rejection of a large number of low-residual noise pixels and preservation of signal pixels. (G) Over n=7 elevational slices (i.e., technical replicates), average apparent SO_2_ estimated without noise masking within ROIs matched in size to the 1.6 mm tube or dilated to 4.8 mm, showing increasing noise bias for the larger ROI due to inclusion of noise pixels in adjacent signal-free water regions (Mean ± SD of absolute SO_2_ difference of 6.0 ± 3.9% between the two ROIs). (H) In unmasked data, proportion of pair-wise SO_2_ comparisons that are statistically significant ($p_{adj}<0.05$, one-way ANOVA followed by Bonferroni-adjusted pairwise comparisons), normalized to the number of significant comparisons at the baseline ROI (1.6 mm). The proportion decreases for increasing ROI size, demonstrating progressive noise bias as the ROI grows into signal-free regions (US/PA background). (I) Application of noise masking to estimates of apparent SO_2_ restores ability to discriminate between ROI-averaged SO_2_ even for large ROIs (Mean ± SD of absolute SO_2_ difference of 2.6 ± 1.7%). (J) With application of the noise mask, the proportion of significant pairwise comparisons is maintained with increasing ROI size.

## 3 Results

### 3.1 Noise characterization

Our experiments (acquired here without energy correction) confirmed that the noise-only PA amplitude was spectrally flat and independent of laser wavelength throughout the image on the Vevo 2100. In the noise-only image, the probability density function (PDF) of the residual was approximately Gaussian (Fig. 2A), allowing the mean μ and standard deviation σ to characterize the distribution. These residual statistics were consistent across acquisitions taken at baseline, 30 min later, and the following day, demonstrating temporal stability of the measure (Fig. 2B).

We next determined the number of temporal frames, or alternatively the number of pixels within a single frame, required for a stable and consistent estimate of residual statistics. Pixel-wise residual means and standard deviations ($\mu_{t},\sigma_{t}$) were computed over 900 temporal frames (equivalent to roughly 180 s) and corresponding spatial estimates ($\mu_{n},\sigma_{n}$) were computed across pixel counts up to 2000 (equivalent to a 1.6 × 2.0 mm ROI) (Fig. 2C). The estimates obtained from the 900-frame acquisition served as the reference for assessing temporal convergence, while progressively larger spatial ROIs were used to assess spatial convergence within a single frame. The normalized temporal standard deviation ($\sigma_{t}/\mu_{t}$) converged rapidly, with its percent difference from the 900-frame reference value falling below 3% by approximately frame 100 (Fig. 2D, inset). Thus, approximately 100 frames were sufficient to obtain a stable temporal estimate of residual statistics relative to the 900-frame reference. Because acquiring even 100 frames entails a lengthy PA acquisition of roughly 20 s, we also evaluated the number of pixels required to achieve comparable results from a single frame. A spatial ROI of approximately 500 pixels was sufficient (Fig. 2E, inset), corresponding to a 0.8 × 1.0 mm ROI in a single frame.

Throughout the field of view, the residual image showed low values in a noise-only acquisition (Fig. 2F). Elevated residuals appeared at the image edges, presumably because those regions are beamformed with fewer transducer elements for lower SNR, and a slight spatial periodicity was attributed to IQ data remodulation during beamforming. The standard deviation image showed a similar pattern, with values remaining low throughout (Fig. 2G), as did the residual threshold map set to μ+3σ per pixel (Fig. 2H). ROIs placed in proximal (red) and distal (orange) regions and across the full image (purple) assessed the impact of ROI selection. The noise-only acquisition thresholded at the mean plus one, three, and five standard deviations, using statistics from each ROI, yielded progressively greater rejection of noise pixels at higher thresholds (Fig. 2I). For smaller, centrally placed ROIs, edge pixels still exceeded the threshold, reflecting their inherently lower SNR. Reflecting this, our noise ROIs for this study were made sufficiently large and placed away from the lateral edges of the field-of-view.

**Fig. 4:**
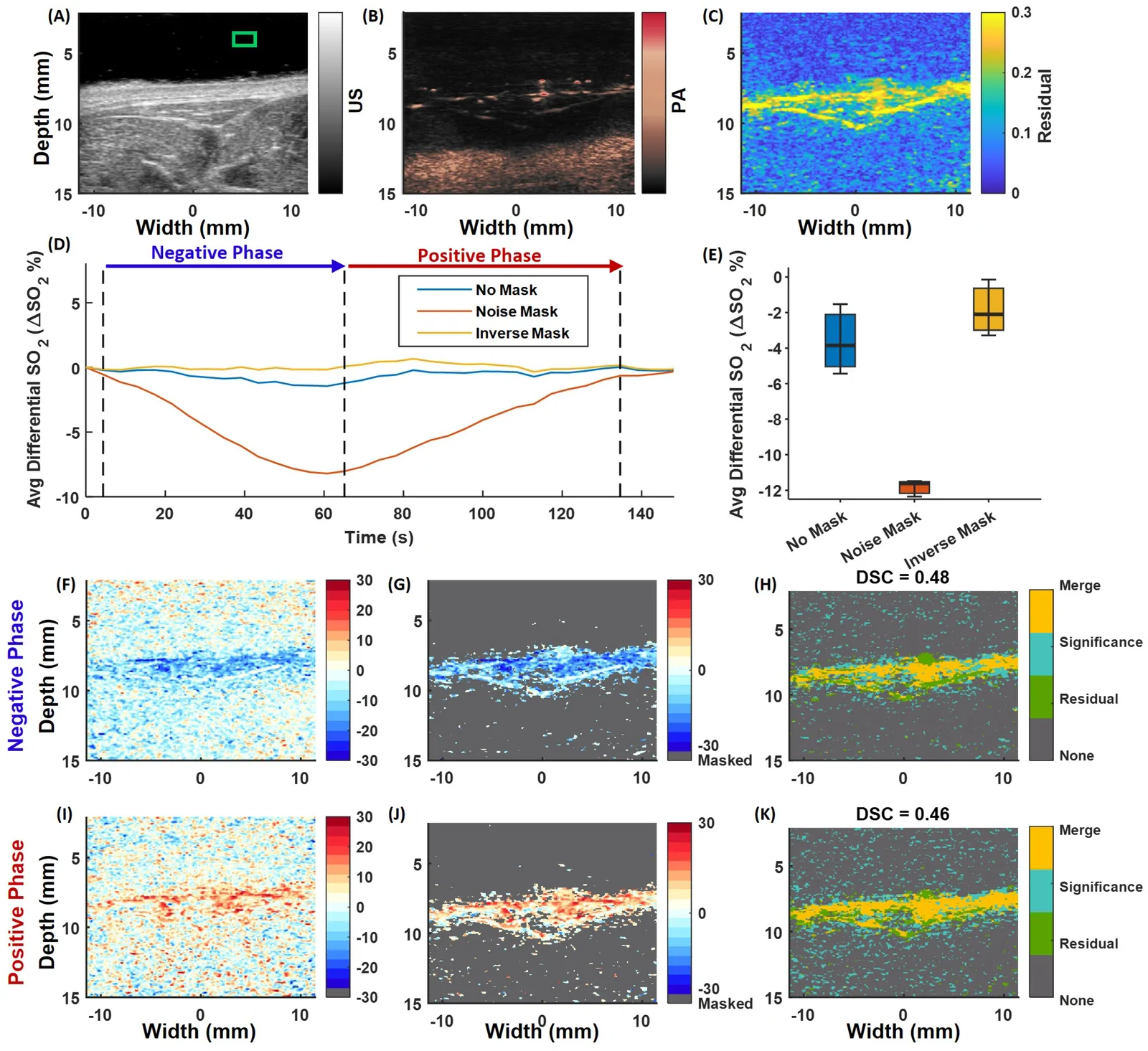
(A) B-mode image of rat skeletal muscle with region for noise estimation marked in green. (B) PA image of rat skeletal muscle at 800 nm. (C) Residual image showing signals with spectra dissimilar to noise in proximal tissue regions. (D) Average ΔSO_2_ over the image with respect to the initial value, showing that masking preserves sensitivity to changes in SO_2_. Onset and end of the oxygen challenge are denoted by dotted vertical lines (n=1 animal with r=3 technical replicates). (E) Average ΔSO_2_ at 60 s after challenge onset was significantly lower for masked (i.e., signal-bearing) pixels (Mean ± SD over three oxygen challenge cycles: −11.8 ± 0.5%) than for the unmasked entire image (−3.6 ± 2.0%) (one-way repeated measures ANOVA followed by Bonferroni-adjusted pairwise comparisons, $p_{adj}<0.040$); the pairwise comparisons between unmasked pixels (−3.6 ± 2.0%) and inverse-masked (i.e., noise-dominated) pixels (−1.8 ± 1.6%), and between masked pixels and inverse-masked pixels, were also significant ($p_{adj}<0.046$, $p_{adj}<0.017$, respectively). (F) ΔSO_2_ from the normoxic to hypoxic state, showing strong decrease in oxygenation in proximal tissue regions, with minimal change in distal regions and standoff due to those regions’ pixels being predominantly noise. (G) Application of the noise mask to ΔSO_2_ preserves regions showing oxygenation change while rejecting noise pixels. (H) Overlay of pixels passing the noise mask and pixels exhibiting statistically-significant change in SO_2_ over oxygen challenge (one-tailed t-test, α=0.05), demonstrating spatial colocalization between the two sets (Dice-Sørensen coefficient of 0.48). (I) ΔSO_2_ from hypoxic to normoxic state, showing a strong increase in oxygenation in proximal tissue regions, with minimal change in distal regions and standoff. (J) Application of the noise mask to ΔSO_2_ preserves regions showing a strong positive ΔSO_2_. (K) Overlay of pixels passing noise mask and pixels exhibiting a statistically significant change (one-tailed t-test, α=0.05), showing moderate spatial colocalization (Dice-Sørensen coefficient of 0.46).

### 3.2 In vitro phantom experiments

To assess the sensitivity improvement from noise masking, we measured SO_2_ values in blood-filled polyethylene tubes. US/PA images of the blood phantom (Fig. 3A, tube outer diameter indicated by white circle) were acquired and the multi-wavelength data were used to calculate the residual (Fig. 3B) and estimate SO_2_ (Fig. 3C). Absolute SO_2_ values are confounded by spectral coloring in the tissue-mimicking phantom, and should therefore be interpreted as apparent estimates. The residual image shows a circular region of elevated residual values within the tube compared to the surrounding region of water with lower residuals, while the apparent SO_2_ image shows elevated values corresponding to blood within the tube, and noise-biased values outside. Applying the mask to the apparent SO_2_ image restricted the analysis to signal-bearing regions, substantially reducing the contribution of noise bias from signal-free regions surrounding the tube (Fig. 3D).

To illustrate the residual calculation, we plotted PA spectra from pixels with known blood signal and known noise-only content alongside the scaled reference noise spectrum $\overset{ˆ}{c}S_{n}$ (Fig. 3E). Noise pixels (blue) closely matched the reference spectrum and yielded a low residual, while signal pixels were spectrally dissimilar and yielded a high residual. The distribution of residual values in the vicinity of the blood-filled tube showed a large peak of low-residual noise pixels and a second peak of higher-residual signal pixels above the threshold (Fig. 3F).

Box-and-whisker plots of apparent SO_2_ estimated within different-sized ROIs without noise masking (Fig. 3G) illustrate how noise bias becomes more pronounced with increasing size (from 1.6 mm to 4.8 mm). We assessed sensitivity using pairwise ΔSO_2_ comparisons with a one-way ANOVA, as ΔSO_2_ is more robust to spectral coloring [29]. The number of significant pairwise comparisons served as a measure of sensitivity to SO_2_ change as a function of ROI size (Fig. 3H, unmasked case). Expanding the ROI beyond the 1.6 mm tube outer diameter in 0.4 mm increments up to 4.8 mm produced a progressive decrease in significant pairs, demonstrating that sensitivity degrades without noise masking as the proportion of included noise pixels grows. Noise masking substantially reduced noise-related bias from larger ROIs (Fig. 3I), and pairwise sensitivity was maintained above 80% even at the largest ROI (Fig. 3J). Finally, we investigated how the choice of noise-estimation region affects residual masking. For the same data, a signal-free standoff region maintained masking performance, a distal region performed slightly worse, and a signal-containing region substantially degraded performance (Fig. S1).

### 3.3 In vivo oxygen challenge experiments

To demonstrate the utility of noise masking when estimating changes in tissue oxygenation *in vivo*, we performed US/PA imaging of the skeletal muscle of an anesthetized rat over multiple oxygen challenge cycles (Fig. 4A/B). The reference noise spectrum from a signal-free region in the gel standoff was used to calculate the residual, which revealed regions of high dissimilarity from noise concentrated at the skin surface and superficial vasculature (Fig. 4C).

We calculated ΔSO_2_ over the course of the oxygen challenge with respect to the baseline value and grouped the oxygen challenge into a negative phase and positive phase (annotated on Fig. 4D; challenge onset marked by vertical dashed line). Taking the average across the entire image yielded only a minor change in tissue oxygen saturation within 60 s of challenge onset (Fig. 4D). This small change reflects the diluting effect of many non-changing noise pixels on the comparatively few pixels with a true SO_2_ response. Noise masking produced a marked improvement in sensitivity to ΔSO_2_, while the inverse set of pixels (i.e., noise-dominated) showed minimal ΔSO_2_, confirming that their inclusion in any averaged estimate degrades sensitivity. Quantitatively, noise masking yielded ΔSO_2_ of up to −11.8 ± 0.5% (mean ± SD) from onset to 60 s into the challenge, compared with −3.6 ± 2.0% and −1.8 ± 1.6% for the unmasked and inverse mask cases, respectively (Fig. 4E). We used a one-way repeated-measures ANOVA with Bonferroni-adjusted pairwise comparisons to assess significance, finding all three pairwise comparisons to be significant ($p_{adj}<0.05$).

We analyzed SO_2_ difference images computed for both negative and positive phases of the oxygen challenge (annotated on Fig. 4D). In both cases, unmasked difference images showed strong ΔSO_2_ concentrated at the skin surface, with the direction of the change consistent with the phase of the oxygen challenge (Fig. 4F/I). Most pixels in the distal and standoff regions showed minimal ΔSO_2_, consistent with their spectra being dominated by noise rather than true physiological signal. Application of the noise mask to ΔSO_2_ concentrated the retained pixels near the tissue surface, consistent with laser fluence and SNR being highest in proximal regions, while rejecting noise-dominated pixels in the distal tissue and standoff (Fig. 4G/J).

We compared the noise mask against pixels exhibiting a statistically significant change in SO_2_ over each phase of the oxygen challenge (one-tailed t-test, α<0.05). In the negative and positive phases, significant pixels were concentrated in proximal regions, and overlaying the significance map with the noise mask revealed spatial colocalization between challenge-responsive and noise-masked pixels (Fig. 4H/K), with Dice-Sørensen coefficients of 0.48 and 0.46, respectively. The moderate DSC values likely reflect false positives among distal and standoff pixels in the t-test—attributable to the inherent variability of SO_2_ estimates from noise-dominated pixels—whereas the noise mask reliably rejected these regions for both phases of the oxygen challenge, demonstrating its advantage over pixel-wise significance testing alone.

We also studied the impact of noise-region placement on masking performance, finding that an alternate proximal region maintained performance, the distal region performed slightly worse, and a signal-bearing region substantially degraded sensitivity to changes in SO_2_ (Fig. S2).

## 4 Discussion

We have presented a pixel-wise noise masking approach that differentiates pixels on the basis of their spectral similarity to noise. Because the residual is computed from the normalized shape of the PA spectrum rather than its absolute amplitude (Fig. 3E), the proposed method may offer complementary advantages to amplitude- or SNR-based masking, particularly when variations in acquisition parameters affect signal amplitude while the spectral shape remains distinguishable from noise. This spectral criterion may also be useful for retaining low-amplitude pixels whose spectra remain dissimilar from the noise reference.

Noise and uncertainty in quantitative PA imaging have been addressed using several alternative strategies. Simple approaches apply fixed signal-amplitude thresholds or percentages of the maximum signal [37], whereas SNR-regularized methods use amplitude and noise information to determine which pixels are retained for analysis [38]. More sophisticated data-driven approaches include confidence estimation for quantitative PA inversion [39], learned spectral decoloring [40], wavelength-flexible SO_2_ estimation [41], and Bayesian deep-learning methods that provide pixel-wise uncertainty estimates [42]. These approaches can address sources of error beyond simple noise exclusion but may require training data, model calibration, and greater computational complexity. In contrast, our noise masking method classifies pixels directly according to their spectral similarity to a measured noise reference, requires no training, and is readily interpretable. However, it addresses noise-related bias rather than other important sources of quantitative error, including fluence variation, spectral coloring, unmixing error, and optical or acoustic artifacts.

Noise bias can substantially affect ROI-based PA biomarker estimates, particularly at depth where reduced fluence and SNR increase the fraction of noise-dominated pixels. Noise masking is well suited to address this challenge in ROI-based quantification of SO_2_ and other spectroscopic biomarkers [8], [43], and may also benefit dynamic PA biomarkers derived from contrast-agent kinetics such as uptake and clearance rates, time to peak, half-life, and area under the curve [44], [45], [46]. In these cases, low SNR at early and late time points can bias kinetic parameter estimates in deep or heterogeneous organs such as the liver or kidneys. The utility of our approach extends to longitudinal and intersubject measurements: changes in tumor size or ROI composition over time alter the proportion of low-SNR pixels and confound longitudinal estimates, while increased epidermal melanin reduces fluence at depth and raises the low-SNR fraction, potentially introducing skin-tone–dependent bias [47], [48]. Although noise masking excludes these noise-dominated pixels, it does not correct melanin-related fluence attenuation or spectral effects, which must be addressed separately in spectral unmixing.

Although spectral coloring in the tissue-mimicking phantom precluded accurate absolute SO_2_ estimation, necessitating use of apparent SO_2_ and ΔSO_2_ for quantitative analysis, the progressive degradation in pairwise sensitivity with increasing ROI size, and its restoration by noise masking, demonstrates the utility of our method independently of this confounding factor. We also note that we computed average apparent SO_2_ and its change over the entire field of view in our *in vivo* experiments rather than focusing on a specific ROI, given the relative tissue homogeneity of skeletal muscle in the absence of a focal region such as a tumor. This choice also avoided potential bias introduced by segmentation itself, a separate challenge in PA imaging discussed elsewhere [49].

Our analysis was based on beamformed PA amplitude data rather than single-channel RF or IQ data, to maintain applicability to a wide range of pre-clinical and clinical systems for which the latter data may not be available. The method is straightforward to implement and computationally efficient; it requires few user-defined choices beyond the selection of a noise ROI, and the use of pixel-wise statistical analysis makes it robust across imaging scenarios. Though demonstrated on the Vevo 2100, the method may be applicable to a greater range of PA systems since electronic noise arises from the receive hardware rather than from laser-tissue interaction and is therefore consistent in character across platforms. The key requirement is that the noise spectrum be consistent in space and time after preprocessing—a condition satisfied by estimating it from the standoff, since the same processing pipeline acts on both signal and noise regions equally.

The method depends on two user-defined choices. The first is the reference region: masking was robust to reasonable changes in the placement of a signal-free standoff region, but performance was slightly reduced when a distal region was used and substantially impaired when the reference region contained PA signal, which altered the reference spectral shape and estimated threshold (Fig. S1, S2). Reference-region selection should therefore be restricted to regions known to be free of signal, and future work should evaluate automated and contamination-tolerant noise-reference estimation. The second user-defined choice is the threshold: in the present data, signal-bearing pixels were well separated from the noise-residual distribution, making μ+3σ a useful compromise between noise rejection and signal retention, though this cutoff is not expected to be universally optimal. A less stringent threshold may be preferable when weak-signal retention is prioritized, or when target spectra closely resemble the noise spectrum—for example, in acquisitions without energy correction where a chromophore’s effective absorption spectrum appears spectrally flat, reducing its spectral difference from the noise reference. The risk is generally low for commonly imaged endogenous absorbers such as oxy/deoxyhemoglobin, collagen, and lipids, which have distinctive spectral features that distinguish them from noise [50], but certain exogenous agents with broadband absorption spectra may pose a challenge [51]. However, it is unlikely that any exogenous spectrum would be identical to noise, particularly when more wavelengths are used for residual calculation.

We note several limitations of our study. We did not perform a direct head-to-head comparison with amplitude- and SNR-based masking; such comparisons will be necessary to determine relative performance under matched threshold-selection criteria. The *in vivo* study also used one animal with three repeated oxygen challenges, which are technical rather than biological replicates, and no independent blood-gas, pulse-oximetry, or local tissue-oxygenation reference was acquired; the experiment therefore demonstrates sensitivity to a controlled perturbation of known direction but does not establish the quantitative accuracy of the measured ΔSO_2_ magnitude. Further work is required for biological replication and external physiological validation. Finally, we used a standard linear least-squares unmixing algorithm with the same four wavelengths for both residual calculation and SO_2_ estimation; this set was chosen for hemoglobin unmixing and fast acquisitions, and was also sufficient for residual calculation, as these wavelengths distinguish HbO_2_ and HHb from noise. Whether fewer wavelengths would suffice for reliable residual calculation, or whether wavelength sets optimized jointly for unmixing and noise discrimination would improve performance, remains an open question. The choice of unmixing algorithm and wavelength selection each substantially influence measurement accuracy in spectroscopic PA imaging [28], [52], [53]; integrating noise masking with advanced unmixing techniques and evaluating wavelength optimization for residual calculation are promising directions for future work.

In summary, we present a residual-based noise masking approach that reduces the contribution of noise-dominated pixels to ROI-based spectroscopic PA biomarker estimation. Using ΔSO_2_ as a representative application, phantom and *in vivo* oxygen-challenge data demonstrate improved sensitivity to biomarker changes after masking. The method does not correct fluence attenuation, spectral coloring, spectral-unmixing error, or other sources of quantitative uncertainty and should therefore be considered complementary to methods addressing these effects. Future work should evaluate performance across additional systems, biological replicates, noise-reference conditions, and biomarkers.

## CRediT authorship contribution statement

**David Qin:** Writing – review & editing, Writing – original draft, Visualization, Validation, Methodology, Investigation, Conceptualization. **Xinyue Huang:** Writing – review & editing, Visualization, Validation, Methodology, Investigation. **Scott J. Schoen Jr.:** Writing – review & editing, Visualization, Validation, Methodology, Investigation. **Stanislav Emelianov:** Writing – review & editing, Supervision, Resources, Project administration, Methodology, Funding acquisition. **Richard R. Bouchard:** Writing – review & editing, Writing – original draft, Supervision, Project administration, Methodology, Funding acquisition, Conceptualization.

## Animal subject

This study was conducted in accordance with the ARRIVE (Animal Research: Reporting of In Vivo Experiments) guidelines.

This study was approved by the Georgia Institute of Technology Institutional Animal Care and Use Committee.

(Approval No. A100865)

## Declaration of generative AI use

The authors used Claude Opus 4.8 for text proofreading and to debug existing code, after which the authors reviewed and edited the text, while code was examined in full and its outputs compared to the original code. The authors take full responsibility for the content of the published article.

## Declaration of competing interest

The authors declare that they have no known competing financial interests or personal relationships that could have appeared to influence the work reported in this paper.

## Data Availability

Data will be made available on request.

## Appendix A Supplementary data

MMC S1
