# Supplementary material for: Improved quantification of photoacoustic biomarkers through residual-based noise masking
Source: Photoacoustics. 2026 Sep 10;51:100880. doi: 10.1016/j.pacs.2026.100880 (PMC13595085; doi:10.1016/j.pacs.2026.100880)
Supplement: Supplementary file 1 [file mmc1.pdf]

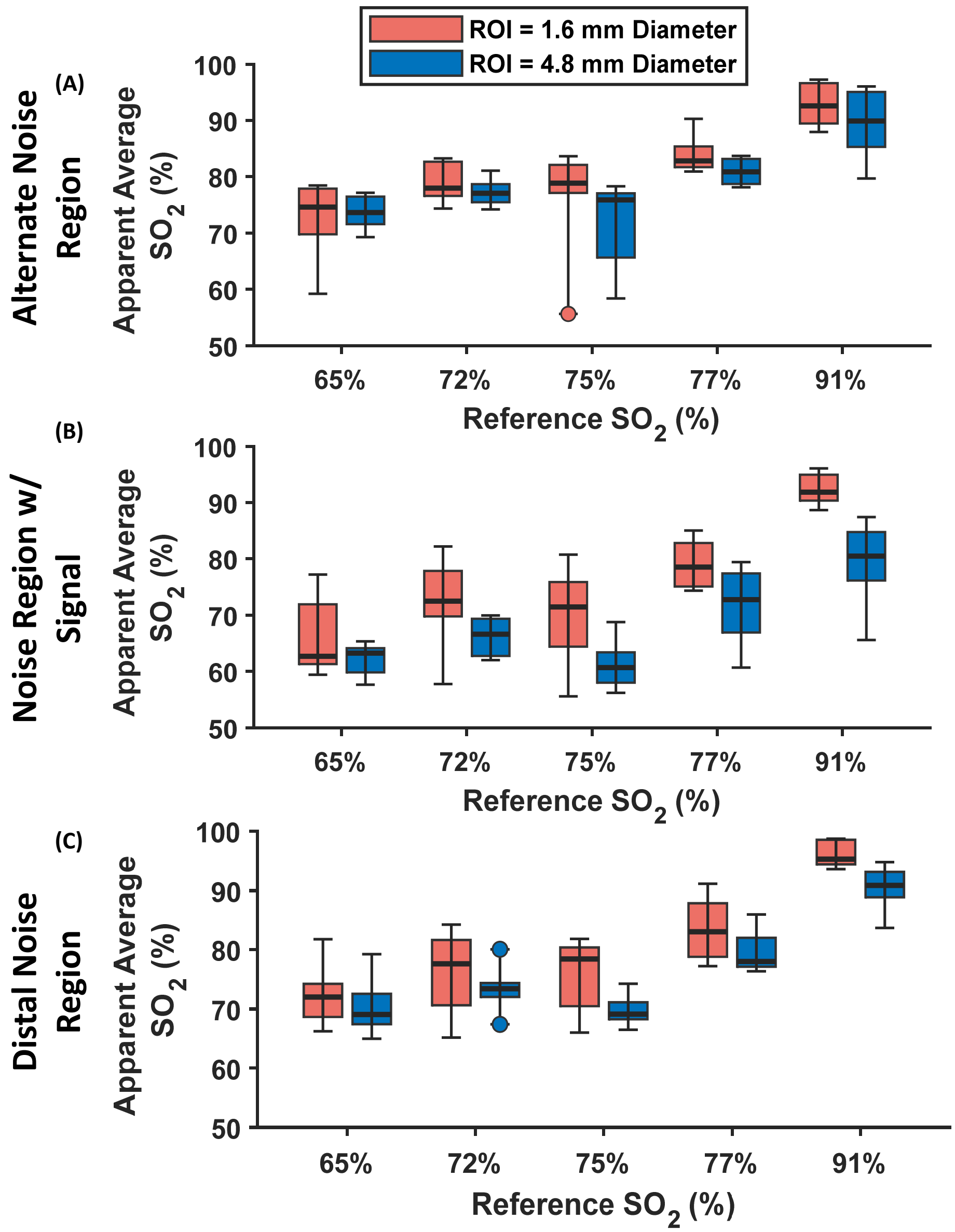

Figure S1: Effect of noise-region selection on SO_2 bias for *in vitro* noise-reference estimation and pixel-wise residual calculation, comparing masking performance for a 1.6-mm-diameter ROI centered on the tube and a dilated 4.8-mm ROI including regions surrounding the tube. (A) A proximal noise region at a different location within the signal-free standoff yields comparable performance (mean ± SD absolute SO_2 difference between the two ROIs: 2.8±1.5%) to the original noise region (2.6±1.7%). (B) Including signal in the noise region substantially degrades masking (7.9±3.3%), while (C) selection of a distal noise region slightly decreases masking performance (4.0±1.8%), resulting in greater noise-induced bias with increasing ROI size.

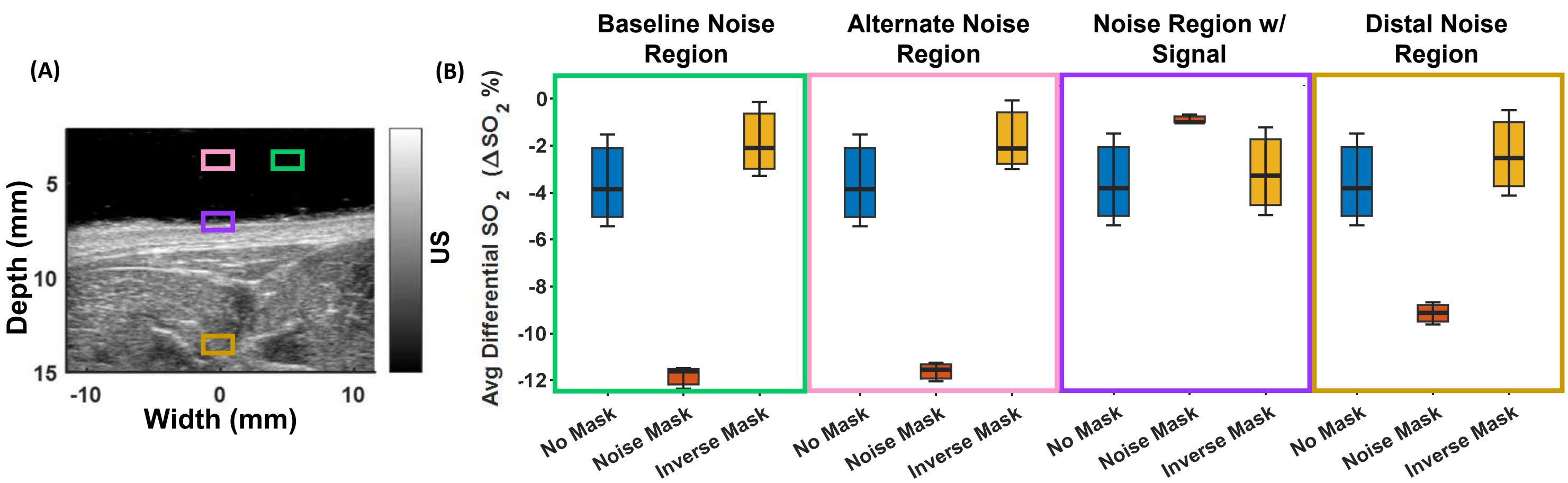

Figure S2: Variation of SO_2 bias with different noise-region selections for *in vivo* noise-reference estimation and pixel-wise residual calculation. (A) In addition to the baseline noise region (green), three noise regions are compared: an alternate location within the signal-free standoff (pink), a region bordering the skin that includes signal (purple), and a distal region within the tissue with low SNR (brown). (B) ΔSO_2 (mean ± SD) for noise masked (i.e., signal-bearing) pixels shows that noise masking is robust to noise-region selection: sensitivity of signal-only pixels is enhanced using baseline noise region (-11.8±0.5%) and an alternate noise region in the signal-free standoff (-11.6±0.4%), and slightly reduced when using the distal noise region (-9.6±0.4%); masking fails when noise is estimated from the signal-containing region at the skin (-0.9±0.2%). For comparison, ΔSO_2 for the unmasked image (i.e., all pixels) was -3.6±2.0%. Average ΔSO_2 of the pixels in the inverse mask (i.e., noise-dominated) was -1.8±1.6%, -1.7±1.5%, -3.2±1.9% and -2.0±1.8%, respectively, for baseline noise region, alternate noise region, noise region w/ signal, and distal noise region.
